# Diterpenoids and Limonoids from the Leaves and Twigs of *Swietenia mahagoni*

**DOI:** 10.1007/s13659-014-0006-6

**Published:** 2014-03-11

**Authors:** Wei-Ming Zhang, Jie-Qing Liu, Yuan-Yuan Deng, Jian-Jun Xia, Zhi-Run Zhang, Zhong-Rong Li, Ming-Hua Qiu

**Affiliations:** 1 State Key Laboratory of Phytochemistry and Plant Resources in West China, Kunming Institute of Botany, Chinese Academy of Sciences, Kunming, 650201 China; 2 University of Chinese Academy of Sciences, Beijing, 100049 China

**Keywords:** Meliaceae, *Swietenia mahagoni*, Diterpenoids, Limonoids

## Abstract

Three new compounds, including two diterpenoids, nemoralisins H and I (**1** and **2**), and a limonoid, 2-methoxy khayseneganin E (**3**), along with four known constituents (**4**–**7**), were isolated from the leaves and twigs of *Swietenia mahagoni*. Their chemical structures were elucidated by means of spectroscopic analysis. The cytotoxities of these isolated constituents were assayed.

**Electronic supplementary material:**

The online version of this article (doi:10.1007/s13659-014-0006-6) contains supplementary material, which is available to authorized users.

## Introduction

*Swietenia mahagoni*, which is an economically important timber tree, has been used as a folk medicine for the treatment of hypertension, diabetes, and malaria [1, 2]. Chemical investigations conducted previously on *S*. *mahagoni* had led to the isolation of various B,D-*seco* limonoids such as mexicanolides, phragmalins, andirobins, gedunins, and rearranged phragmalins [3–7]. Modern pharmacological studies demonstrated that limonoids from *Swietenia* displayed insecticide, antitumor, antibacterial, antidiabetic, and antidyslipidemic activities [8–12].

**Fig. 1:**
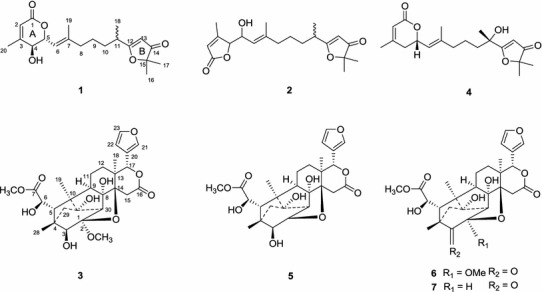
The structure of compounds **1**–**7**

With the aim of searching for structurally unique and bioactive chemical constituents, we isolated the leaves and twigs of *S*. *mahagoni* and gained two new diterpenoids (**1** and **2**), one new limonoid (**3**) and four known compounds (**4**–**7**) (Fig. 1). In this paper, we reported the isolation and structural elucidation of new compounds, as well as cytotoxicities of all the compounds against five human cancer cell lines.

## Results and Discussion

The air-dried powder of leaves and twigs of *S. mahagoni* was extracted with 70 % aqueous acetone at room temperature three times to give the residue, which was then partitioned between EtOAc and water to get the EtOAc**-**soluble fraction. Then, three new constituents together with four known compounds were acquired by a series of chromatographic methods. Herein, we described the isolation and structural elucidation of these new compounds.

**Fig. 2:**
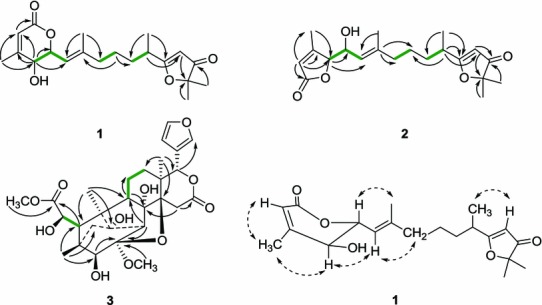
^1^H-^1^H COSY () and key HMBC () correlations of **1**–**3** and key ROESY () correlations of **1**

Compound **1**, colorless oil, was assigned a molecular formula C_20_H_28_O_5_ according to its positive HREIMS peak at *m/z* 348.1932 [M]^+^ (calcd for 348.1937), suggesting seven degrees of unsaturation. The IR spectrum revealed characteristic bands corresponding to hydroxyl (3,434 cm^−1^), *α,β*-unsaturated-*δ*-lactone (1,761 cm^−1^) and double-bond (1,639 cm^−1^) groups. The ^1^H NMR spectrum of **1** (Table 1) showed the presence of three typical olefinic protons *δ*_H_ (5.80, s**-**like; 5.42, s; 5.26, dd, *J* = 8.7, 1.2 Hz), three methyl singlets at *δ*_H_ 1.35 (6H), 2.05 (3H) and two methyl doublets at *δ*_H_ (1.23, *J* = 7.0 Hz; 1.75, *J* = 1.2 Hz). The ^13^C DEPT displayed five methyls, three methylenes, six methines, and six quaternary carbons (Table 1). 1D-NMR data of **1** exhibited high resemblance with nemoralisin A isolated from *Aphanamixis grandifolia* (Meliaceae) [13], and they showed the same molecular formula and the similar skeletal structure (*α,β*-unsaturated ketone was connected with one *α,β*-unsaturated *δ***-**lactone by an aliphatic chain). The only difference between **1** and nemoralisin A was that the hydroxyl group located at C-4 in **1** but at C-8 in nemoralisin A. And this was confirmed by HMBC correlations of H-4 (*δ*_H_ 4.04, d, *J* = 7.2 Hz) with C-2 (*δ*_C_ 117.1), C-3 (*δ*_C_ 162.6), C-5 (*δ*_C_ 80.5), C-6 (*δ*_C_ 121.9), and of Me-20 (*δ*_H_ 2.05) with C-4 (*δ*_C_ 69.7), and together with by the ^1^H-^1^H COSY cross-peaks between H-4 and H-5 (*δ*_H_ 4.99, d, *J* = 8.7, 7.2 Hz) (Fig. 2)

.

**Table 1:** ^1^H and ^13^C-NMR spectroscopic data of compounds **1** and **2** (*δ* in ppm)

Pos.	1^a^		2^b^	
	*δ*_H_ (*J* in Hz)	*δ*_C_	*δ*_H_ (*J* in Hz)	*δ*_C_
1		166.3 s		176.3 s
2	5.80 (s-like)	117.1 d	5.83 (s)	118.9 d
3		162.6 s		170.7 s
4	4.04 (d, 7.2)	69.7 d	4.89 (overlap)	89.3 d
5	4.99 (dd, 8.7, 7.2)	80.5 d	4.70 (dd, 8.8, 3.1)	68.2 d
6	5.26 (dd, 8.7, 1.2)	121.9 d	5.20 (d, 8.8)	124.3 d
7		145.4 s		142.1 s
8	2.10 (m), 2.10 (m)	40.5 t	2.03 (m), 2.03 (m)	40.7 t
9	1.49 (m), 1.49 (m)	25.9 t	1.44 (m), 1.44 (m)	26.5 t
10	1.64 (m), 1.52 (m)	34.7 t	1.60 (m), 1.49 (m)	35.0 t
11	2.71 (m)	36.9 d	2.68 (m)	37.3 d
12		198.9 s		199.5 s
13	5.42 (s)	100.7 d	5.41 (s)	101.1 d
14		210.3 s		210.7 s
15		89.9 s		90.3 s
16	1.35 (s)	23.3 q	1.32 (s)	23.5 q
17	1.35 (s)	23.3 q	1.32 (s)	23.5 q
18	1.23 (d, 7.0)	18.3 q	1.20 (d, 6.9)	18.4 q
19	1.75 (d, 1.2)	17.1 q	1.68 (s)	17.0 q
20	2.05 (s)	19.9 q	2.13 (s)	15.0 q

^a^Recorded in CD_3_OD and CDCl_3_; ^1^H and ^13^C-DEPT Recorded at 600, 150 MHz

^b^Recorded in CD_3_OD, ^1^H and ^13^C-DEPT Recorded at 500, 125 MHz

In the ROSEY spectrum, the strong correlation from H-6 (*δ*_H_ 5.26, dd, *J* = 8.7, 1.2 Hz) to H-8 (*δ*_H_ 2.10, m) inferred *E*-geometry of the Δ^6^ double bond. This was confirmed by the upfield resonance of vinylic Me-19 group at *δ*_C_ 17.1 [14]. The ROESY correlations of H-4/H-6 and H-5/Me-19 (*δ*_H_ 1.75, d, *J* = 1.2 Hz) suggested that H-4 and H-5 was *trans* oriented. However, the absolute configuration was not determined on the basis of 1D and 2D NMR data. Accordingly, compound **1** was established and named as nemoralisin H.

Compound **2** was isolated as colorless oil. The molecular formula of C_20_H_28_O_5_ was determined by the positive ion peak at *m/z* 348.1927 [M]^+^ (calcd for 348.1937) in the HREIMS. Extensive analysis of the 1D-NMR spectroscopic data (Table 1) of **2** exhibited a close resemblance with nemoralisin [14]. However, downfield shifts of the lactone carbon (*δ*_C_ 176.3), the *β*-*sp*^2^ carbon (*δ*_C_ 170.7), and the oxygenated methine (*δ*_C_ 89.3) were observed in the ^13^C DEPT spectrum of **2**, which implied the *α,β*-unsaturated *δ***-**lactone (ring A) in nemoralisin was replaced by an *α,β*-unsaturated *γ*-lactone in **2**. This deduction was confirmed by the observed HMBC correlations of H-4 (*δ*_H_ 4.89) with C-1 (*δ*_C_ 176.3), C-2 (*δ*_C_ 118.9), C-3 (*δ*_C_ 170.7), C-6 (*δ*_C_ 124.3), and of Me-20 (*δ*_H_ 2.13) with C-4 (*δ*_C_ 89.3) (Fig. 2). Meanwhile, on the basis of ^1^H-^1^H COSY correlations of H-4/H-5/H-6, a hydroxyl group at C-5 and Δ^6^ double bond were established. The ROESY correlations of H-6 (*δ*_H_ 5.20, d, *J* = 8.8 Hz) with H-8 (*δ*_H_ 2.03, m) inferred *E*-geometry of the Δ^6^ double bond. Therefore, the structure of **2** was determined and gave the name nemoralisin I.

Compound **3**, white amorphous powder, was found to possess the molecular formula of C_28_H_36_O_11_ as inferred by HREIMS peak at *m/z* 548.2250 [M]^+^ (calcd for 548.2258). The 1D NMR data (Table 2) of **3** indicated that it was a phragmalin-type limonoid and similar with khayseneganin E [15–17]. However, a methoxy in **3** replaced the hydroxyl group at C-2 in khayseneganin E, which was supported by HMBC correlation between OMe (*δ*_H_ 3.68) and the ketal carbon (*δ*_C_ 106.9) (Fig. 2). The relative configuration of **3** was determined by analysis of the ROESY spectrum, in which correlations of H-5/H-6, H-5/H-12*β*, H-12*β*/H-17, and H-6/Me-28 suggested that these groups are all *β*-oriented. In addition, Me-19, Me-18, H-9, and H-3 were assigned as *α*-oriented on the basis of the ROESY correlations between Me-19/H-9 and between H_2_-29/H-3, Me-18/H-3, suggesting that OH-3 is *β*-oriented. Consequently, the structure of **3** was determined as shown, named 2**-**methoxy khayseneganin E.

**Table 2:** ^1^H and ^13^C-NMR spectroscopic data of compound **3** (*δ* in ppm)

Pos.	*δ*_H_ (*J* in Hz)	*δ*_C_	Pos.	*δ*_H_ (*J* in Hz)	*δ*_C_
1		85.1 s	15	3.82 (d, 18.3), 4.20 (d, 18.3)	37.8 t
2		106.9 s	16		172.4 s
3	3.78 (d, 5.9)	84.7 d	17	6.26 (s)	81.7 d
4		43.7 s	18	1.39 (s).	16.3 q
5	3.50 (d, 8.4)	41.4 d	19	1.89 (s)	19.2 q
6	4.74 (d, 8.4)	72.3 d	20		122.7 s
7		176.7 s	21	7.55 (br.s)	142.1 d
8		87.9 s	22	6.54 (br.s)	111.5 d
9	2.96 (d, 9.2)	57.3 d	23	7.62 (br.s)	143.7 d
10		61.3 s	28	1.39 (s)	20.6 q
11	2.11 (m), 2.51 (m)	17.7 t	29	1.94 (d, 11.8), 2.39 (d, 11.8)	46.0 t
12	1.22 (m), 2.51 (m)	28.3 t	30	3.55 (s)	74.5 d
13		38.9 s	OMe-7	3.73 (s)	52.4 q
14		84.7 s	OMe-2	3.68 (s)	51.2 q

Recorded in C_5_D_5_N; ^1^H and ^13^C-DEPT Recorded at 600, 150 MHz

Four known constituents: nemoralisin C (**4**) [13], khayanolide B (**5**) [18], khayseneganin G (**6**) [17], and deacetylkhayanolide E (**7**) [19], were identified by the comparison of their spectroscopic data with those reported in the literature.

Compounds **1**–**7** were tested for in vitro inhibitory activities against HL-60, SMMC-7721, A549, MCF-7 and SW480 human tumor cell lines. All the tested samples showed no activities against the mentioned cell lines with IC_50_ > 40 μM. Much to our delight, Liu J. et al. [20] reported that aphadilactones A−D, which can be considered as the dimers of diterpenoid compounds (**1**, **2** and **4**) showed potent and selective inhibition against the diacylglycerol *o*-acyltransferase-1 (DGAT-1) enzyme, and are the strongest natural DGAT-1 inhibitors discovered to date. So emphasis can be laid on this class of compounds in our future search of potential DGAT-1 inhibitors.

## Experiment Section

### General Experimental Procedures

Optical rotations were obtained with a Jasco P-1020 polarimeter. UV (in MeOH) and IR (in CHCl_3_) spectra were measured on Shimadzu UV-2401 PC spectrophotometer and Bruker Tensor-27 infrared spectrophotometer, respectively. ESIMS spectra were recorded on an API QSTAR Pulsar spectrometer. EIMS and HREIMS were performed on a Waters Autospec Premier P776. 1D and 2D NMR spectra were recorded on Bruker DRX-500 and Bruker Avance III-600 MHz spectrometers. Chemical shifts (*δ*) were expressed in *ppm* with reference to the TMS resonance. Semi-preparative HPLC studies were carried out on an Agilent 1100 liquid chromatograph with a Zorbax SB-C18 (9.4 mm × 25 cm) column. Column chromatography was performed using Silica gel [(200–300) mesh, Qingdao Marine Chemical, Inc, Qingdao, China]. Fractions were monitored by TLC and spots were visualized by heating the silica gel plates sprayed with 10 % H_2_SO_4_ in EtOH. Lichroprep RP-18 [(40–63) μm, Merck] and Sephadex LH-20 [(20–150) μm, Pharmacia] were also used for column chromatography.

### Plant Material

The leaves and twigs of *S. mahagoni* were collected from Xishuangbanna, Yunnan Province, China. A voucher sample has been deposited in the State Key Laboratory of Phytochemistry and Plant Resources in West China, Kunming Institute of Botany, Chinese Academy of Sciences.

### Extraction and Isolation

The air-dried powdered leaves and twigs of *S. mahagoni* (10 kg) were extracted with 70 % aqueous acetone (each 30 L, 3 days) at room temperature three times to give a dark green residue (650 g), which was then partitioned between EtOAc and water to give the EtOAc-soluble fraction (120 g). The EtOAc extract was chromatographed by silica gel column eluted with CHCl_3_–MeOH as a gradient (100:1, 50:1, 20:1, 5:1) to afford four fractions. The CHCl_3_–MeOH (100:1) portion was evaporated to obtain a residue (20 g), which was subjected to silica gel chromatograph column with petroleum ether–EtOAc (10:1, 6:1, 3:1, 1:1) as elution, to give fractions (A, B, C, D). Fraction B was further subjected to RP-18 chromatograph column, eluting with MeOH–H_2_O (50:50, 65:35, 80:20) to afford subfraction (E), which was then purified by semi-preparation HPLC with MeCN–H_2_O (50:50) to give compound **3** (6 mg), **5** (12 mg), **6** (10 mg), **7** (15 mg). Fraction C was subjected to silica gel chromatograph column with petroleum ether–EtOAc (8:1, 5:1, 3:1, 1:1) as elution, to give fractions F, which was successively subjected to RP-18, Sephadex LH-20 and semi-preparation HPLC, compound **1** (1.5 mg), **2** (1.3 mg), and **4** (6 mg) were obtained.

### Nemoralisin H (**1**)

Colorless oil; [*α*]_D_^25^ − 0.48 (*c* 0.11, MeOH); UV (MeOH) *λ*_max_ (log *ε*) 202 (3.75), 261(3.37) nm; IR (KBr) *ν*_max_ 3434, 2976, 2930, 1761, 1639, 1585, 1457, 1383, 1270, 1177, 1088, and 1036 cm^−1^, ^1^H and ^13^C-DEPT data see Table 1; EIMS *m/z* 371 [M + Na]^+^; HREIMS *m/z* 348.1932 (calcd for C_20_H_28_O_5_ [M]^+^, 348.1937).

### Nemoralisin I (**2**)

Colorless oil; [*α*]_D_^25^ − 14.3 (*c* 0.18, MeOH); UV (MeOH) *λ*_max_ (log *ε*) 204 (3.87), 261(3.70) nm; IR (KBr) *ν*_max_ 3432, 2977, 2933, 1761, 1698, 1584, 1458, 1383, 1299, 1267, 1177, 1034 and 938 cm^−1^, ^1^H and ^13^C-DEPT data see Table 1; ESIMS *m/z* 371 [M + Na]^+^; HREIMS *m/z* 348.1927 (calcd for C_20_H_28_O_5_ [M]^+^, 348.1937).

### 2-Methoxy khayseneganin E (**3**)

White amorphous powder; [*α*]_D_^18^ + 26.3 (*c* 0.03, MeOH); UV (MeOH) *λ*_max_(log *ε*) 209 (3.83) nm; IR (KBr) *ν*_max_ 3440, 2952, 1727, 1632, 1462, 1441, 1387, 1280, 1245, 1159, 1053, 1024, and 600 cm^−1^, ESIMS *m/z* 571 [M + Na]^+^; HREIMS *m/z* 548.2250 (calcd for C_28_H_36_O_11_ [M]^+^, 548.2258).

### Cytotoxicity Assay

The cytotoxic activities of compounds **1**–**7** against HL-60, SMMC-7721, A549, MCF-7 and SW480 cell lines were determined by the MTT method [21].

## Electronic supplementary material

Below is the link to the electronic supplementary material.

Supplementary material 1 (DOC 830 kb)
