# Supplementary material for: Diterpenoids and Limonoids from the Leaves and Twigs of Swietenia mahagoni
Source: Nat Prod Bioprospect. 2014 Mar 11;4(1):53–7. doi: 10.1007/s13659-014-0006-6 (PMC3956973; doi:10.1007/s13659-014-0006-6)
Supplement: Supplementary file 1 — Supplementary material 1 (DOC 830 kb) [file 13659_2014_6_MOESM1_ESM.doc]

Supporting Information Available

**Diterpenoids and Limonoids from the Leaves and Twigs of *Swietenia mahagoni***

Wei-Ming ZHANG,^a,b^ Jie-Qing LIU,^a^ Yuan-Yuan DENG,^a,b^ Jian-Jun XIA,^a^ Zhi-Run ZHANG,^a^ Zhong-Rong LI,^a^ Ming-Hua QIU^a,b^*

^a^State Key Laboratory of Phytochemistry and Plant Resources in West China, Kunming Institute of Botany, Chinese Academy of Sciences, Kunming 650201, China

^b^University of Chinese Academy of Sciences, Beijing 100049, China

**Correspondence**

***MingHua Qiu***

State Key Laboratory of Phytochemistry and Plant Resources in West China

Kunming Institute of Botany, Chinese Academy of Sciences

132 LanHei Road

Kunming 650204, Yunnan, P. R. China.

Tel: +86-871-65223327

Fax: +86-871-65223255

Email address: [mhchiu@mail.kib.ac.cn](mailto:mhchiu@mail.kib.ac.cn)

**S 1.** ^1^H NMR spectrum of compound **1** (methanol-*d*_4_ and chloroform-*d*, 600 MHz).

**S 2.** ^13^C NMR spectrum of compound **1** (methanol-*d*_4_ and chloroform-*d*, 150 MHz).

**S 3.** HMBC spectrum of compound **1**.

**S 4.** COSY spectrum of compound **1**.

**S 5.** HSQC spectrum of compound **1**.

**S 6.** ROESY spectrum of compound **1**.

**S 7.** ^1^H NMR spectrum of compound **2** (methanol-*d*_4_, 500 MHz).

**S 8.** ^13^C NMR spectrum of compound **2** (methanol-*d*_4_, 125 MHz).

**S 9.** HMBC spectrum of compound **2**.

**S 10.** COSY spectrum of compound **2**.

**S 11.** HSQC spectrum of compound **2**.

**S 12.** ROESY spectrum of compound **2**.

**S 13.** ^1^H NMR spectrum of compound **3** (Pridine-*d*_5_, 600 MHz).

**S 14.** ^13^C NMR spectrum of compound **3** (Pridine-*d*_5_, 150 MHz).

**S 15.** HMBC spectrum of compound **3**.

**S 16.** COSY spectrum of compound **3**.

**S 17.** HSQC spectrum of compound **3**.

**S 18.** ROESY spectrum of compound **3**.

Compound **1**

**S 1.** ^1^H NMR spectrum of compound **1** (methanol-*d*_4_ and chloroform-*d*, 600 MHz).

**S 2.** ^13^C NMR spectrum of compound **1** (methanol-*d*_4_ and chloroform-*d*, 150 MHz).

**S 3.** HMBC spectrum of compound **1**.

**S 4.** COSY spectrum of compound **1**.

**S 5.** HSQC spectrum of compound **1**.

**S 6.** ROESY spectrum of compound **1**.

Compound **2**

**S 7.** ^1^H NMR spectrum of compound **2** (methanol-*d*_4_, 500 MHz).

**S 8.** ^13^C NMR spectrum of compound **2** (methanol-*d*_4_, 125 MHz).

**S 9.** HMBC spectrum of compound **2**.

**S 10.** COSY spectrum of compound **2**.

**S 11.** HSQC spectrum of compound **2**.

**S 12.** ROESY spectrum of compound **2**

Compound **3**

**S 13.** ^1^H NMR spectrum of compound **3** (Pyridine-*d*_5_, 600 MHz).

**S 14.** ^13^C NMR spectrum of compound **3** (Pyridine-*d*_5_, 150 MHz).

**S 15.** HMBC spectrum of compound **3**

**S 16.** COSY spectrum of compound **3**

**S 17.** HSQC spectrum of compound **3**

**S 18.** ROESY spectrum of compound **3**
